# Assessment of SNPs for linkage mapping in *Eucalyptus*: construction of a consensus SNP/microsatellite map from two unrelated pedigrees

**DOI:** 10.1186/1753-6561-5-S7-P31

**Published:** 2011-09-13

**Authors:** Bruno Marco Lima, Orzenil B Silva-Junior, Danielle A Faria, Eva MC Mamani, Georgios J Pappas, Dario Grattapaglia

**Affiliations:** 1 EMBRAPA Genetic Resources and Biotechnology, EPqB 70770-910, Brazilia, DF, Brazil / Department of Genetics, University of São Paulo - ESALQ/USP, Brazil; 2 EMBRAPA Genetic Resources and Biotechnology, EPqB 70770-910, Brazilia, DF, Brazil; 3 EMBRAPA Genetic Resources and Biotechnology, EPqB 70770-910, Brazilia, DF, Brazil /Genomic Sciences Program, Catholic University of Brazilia, SGAN 916 Brazilia, DF, Brazil

## Background

High throughput genotyping of SNP (Single Nucleotide Polymorphisms) based markers has been developed for an increasing number of plant and animal species. In forest trees large scale SNP development has been approached mainly by amplicon resequencing targeting specific genes for association genetics studies. This approach, although successfully employed in conifers aided by the use of haploid tissue, is technically laborious in diploids because of the very high levels of nucleotide and indel diversity in highly heterozygous tree genomes. Direct SNP development from large *in silico* sequence resources developed by next-generation sequencing is now a very efficient approach for SNP development in forest trees. We have recently developed a first set of 768 SNPs assayed by the Golden Gate Genotyping Technology for the highly heterozygous genome of Eucalyptus from a mixed Sanger/454 database [1]. We saw that a careful sequence quality assessment and the application of stringent constraints on the SNP surrounding sequences have a significant impact on SNP genotyping performance and polymorphism. With the exception of 72 SNPs specifically selected in 20 candidate genes putatively associated with relevant wood phenotypes, all remaining validated SNPs were randomly picked based solely on in silico quality. In this study we wanted to position these SNPs relative to microsatellites and assess their information content for linkage map construction. To enhance our ability of mapping SNPs we employed two eucalyptus full-sib families involving four different Eucalyptus species.

## Material and methods

Two inter-specific segregating populations of *Eucalyptus*, population IP (*E. grandis* x *E. urophylla*) and DGUGL [(*E. dunnii* x *E. grandis*) x (*E. urophylla* x *E. globulus*)] were used for linkage analysis and map construction. DNA was extracted through CTAB and picogreen quantified. The DNA was used for SNP genotyping on an Illumina BeadStation 500 GX. SNP data were analyzed using GenomeStudio V2009.1 and a GeneTrain score cutoff of 0.25 and call rate ≥0.95 were initially applied to the whole dataset. After that, every single SNP was manually checked for genotyping failures and potential calling errors that would bias the overall analysis and segregation ratios. Linkage analysis, individual and consensus map construction for both segregating populations was performed using Joinmap, v. 3.0 [2].

## Results

The proportion of informative segregating SNPs out of the 768 assayed were similar in the two mapping populations: 215 SNPs in DGUGL (28%) and 239 in IP (31%). SNPs were mapped on top of existing microsatellite maps. The linkage map of the IP population with 409 markers on the 11 expected linkage groups had 215 microsatellites and 194 SNPs, with an observed length of 1,581.3 cM and average distance between markers of 3.9 cM. The DGUGL map also had 11 linkage groups and 430 markers being 236 microsatellites and 194 SNPs with an estimated length of 1,252.4 cM and average distance of 2.9 cM. The consensus map, constructed using both segregating populations included 624 unique markers, of which 320 were microsatellites and 304 SNPs with 1,451.4 cM and average distance of 2.3 cM. The proportion of segregating SNPs in each population individually (28 to 31%) was consistent with a within-species level observed heterozygosity of ~50% for this set of 768 SNPs (Grattapaglia et al. 2011), reminding that none of these parents were used in the generation of the EST database wherefrom these SNP were derived. The rate of mappable SNPs was enhanced to almost 40% (304 SNPs mapped out of 768) by using two mapping populations suggesting that by sampling more full-sib families it should be possible to map most if not all SNPs in this panel. No evident clustering of SNPs was observed suggesting that these SNPs are randomly distributed in the Eucalyptus genome.

## Conclusions

This study shows that large numbers of informative SNPs can be developed directly from in silico sequence databases involving unrelated individuals to the parents of mapping populations. Evidently, by being biallelic, SNPs will be less efficient than multiallelic microsatellites for linkage mapping purposes. This drawback however is clearly compensated by the much higher throughput, automation and lower cost of SNP genotyping. While this is the first reported sizeable scale SNP mapping effort in Eucalyptus, a larger number of informative SNPs mapped at regular intervals will be necessary for broader applications. For example, to implement Genomic Selection in most Eucalyptus breeding programs some 4,500 to 6,000 informative SNPs will be necessary to provide a marker density of 3 to 5 markers/centiMorgan and reach selection accuracies above 70% [3]. This would require a SNP panel of some 9,000 to 12,000 SNPs. While this goal is fully achievable, per sample genotyping cost issues, however, will have to be considered before larger scale SNP developments are undertaken in Eucalyptus. Benchmarking the Golden Gate or Infinium genotyping technologies against other high throughput systems such as DArT and emerging genotyping-by-sequencing methods will eventually define how SNP variants be will assayed in Eucalyptus and for that matter in several other highly heterozygous forest tree species.

**Financial support.** Brazilian Ministry of Science and Technology (CNPq Grant 577047-2008-6), FAP-DF NEXTREE Grant 193.000.570/2009 and EMBRAPA Macroprogram 2 project grant 02.07.01.004.
